# Senior emergency medical care students’ readiness and confidence to practice paediatric pain management

**DOI:** 10.4102/hsag.v31i0.3421

**Published:** 2026-08-12

**Authors:** Suzan-Lynn Smit, Dirk Bester, Ryan E. Matthews

**Affiliations:** 1 Department of Emergency Medical Sciences, Faculty of Health and Wellness Sciences, Cape Peninsula University of Technology, Cape Town, South Africa

**Keywords:** paediatric pain, emergency medical services, prehospital emergency care, health professions education, student readiness

## Abstract

**Background:**

Paediatric pain is frequently under-managed in prehospital care despite the known consequences. Practitioner-related factors, during transition from student to independent practice, influence pain management decisions.

**Aim:**

This study aimed to explore and describe the elements influencing senior undergraduate emergency medical care students’ self-reported attitudes, perceived readiness to practise and confidence in managing paediatric pain in South Africa and to identify self-reported barriers and enablers shaping their approach to paediatric pain management.

**Setting:**

The study was conducted among exit-level emergency medical care students at a South African university.

**Methods:**

A qualitative, descriptive and exploratory design was employed. Using purposive sampling, semi-structured interviews were conducted with ten final-year Emergency Medical Care students registered at a South African university within two months of graduation. Interviews were audio-recorded, transcribed verbatim and analysed inductively using reflexive thematic analysis.

**Results:**

The authors generated two themes. Theme one: Attitudinal dissonance, reflected a disconnect between beliefs about paediatric pain management and confidence in practice. Scepticism towards pain expression, fear of harm and protocol reliance contributed to practice hesitancy. Theme two: Fear and uncertainty, highlighted perceived educational gaps and limited clinical exposure, which reduced participants’ perceived practice readiness.

**Conclusion:**

Emergency medical care students’ self-reported paediatric pain management is shaped by the interaction of attitudes, emotional responses and educational structures within prehospital training.

**Contribution:**

The study provides South African prehospital education-specific insight into factors influencing paediatric pain management and supports the need for curriculum refinement and enhanced learning and teaching methods to improve student preparedness and patient care.

## Introduction

Despite increasing awareness of the importance of pain management, pain remains inadequately assessed and treated in the prehospital environment, with paediatric patients particularly vulnerable to oligoanalgesia (Whitley et al. 2021). Inadequately managed pain in children has been shown to heighten anxiety, interfere with emergency care and contribute to both short- and long-term physiological and psychological consequences (Schug et al. 2020). South African Emergency Medical Services (EMS) may encounter paediatrics in pain in as many as 15.2% of cases, underscoring the significance of pain management in this group (Abdullah, Majiet & Sobuwa 2024).

Factors that influence paediatric pain management may be categorised as environmental factors or organisational factors), practitioner factors and patient factors (Downs, Carey & Mold 2022; Handyside et al. 2021). Environmental and/or organisational factors can include the presence or absence of parents/legal guardians and the support of senior health practitioners (Jemebere, Bekele & Yohannis 2020). Patient factors include the developmental stage of the child, emotional responses and possibly uncooperative behaviour (Alabdulaziz et al. 2024; Alshehri, Levett-Jones & Pich 2024).

Practitioner-based factors are related to the practitioners’ thoughts, education or any other factor, which pertain to the preparation and competence of practitioners, or their willingness to act in professional situations (Wuni et al. 2020). Practitioner factors include attitudes, which influence the action, or inaction, during professional situations. Attitudes act as an enabler or barrier to action and impact the way information, such as professional knowledge, is processed and received (Ajzen 2014).

Attitudes undergo significant development and evolution during undergraduate education and training, where structured theoretical learning, workplace placements and exposure to professionals and professional decision-making shape students’ perceptions and beliefs (CHE 2023). Attitude development is supported by continuous reflection, peer support and effective mentorship, all of which are important in cultivating personal beliefs (Ericsson et al. 2024).

Knowledge is a critical practitioner-based factor influencing paediatric pain management, shaping clinicians’ ability to recognise, assess and treat pain effectively. Evidence suggests that limited formal education, insufficient clinical exposure to paediatric patients and challenges in applying pain assessment tools contribute to suboptimal knowledge among prehospital providers (Shamsi 2025). Gaps in understanding of the analgesic options, developmental considerations and communication strategies hinder clinicians’ confidence and decision making, ultimately affecting quality of care (Whitley et al. 2021). Strengthening knowledge through structured education, simulation-based training and reinforcing clinical guidelines has been shown to enhance practitioner preparedness and support more consistent, evidence-based paediatric pain management (Forster et al. 2025; Gai et al. 2020; Kim, Song & Kim 2023).

Knowledge, attitudes and practice are conceptually related. Attitudes, in particular, are recognised as an important part of the development and improvement of clinical pain management practices (Afzal et al. 2021).

As reflected in the Theory of Planned Behaviour, knowledge and attitudes influence behavioural intentions and, in turn, practice, providing a useful lens through which to interpret clinical decision making (Ajzen 2014).

Saudi Arabian emergency care students were found to have attitudes and knowledge that are unfavourable for the provision of appropriate and evidence-based pain management (AlRazeeni 2021). A Ghanaian Paediatric Nurse Knowledge, Attitude, and Practice Survey (PNKAS) found that nursing students scored below 50% on paediatric pain knowledge, while their attitudes towards pain management were also rated as poor (Kusi Amponsah et al. 2019). Egyptian undergraduate nursing students were also reported to have significant knowledge gaps and ‘inappropriate’ attitudes towards paediatric pain management (Gadallah, Hassan & Shargawy 2017).

A South African Knowledge, Attitudes and Practices (KAP) survey conducted among EMS personnel of all qualifications also highlighted inappropriate attitudes towards prehospital pain management (including in paediatrics) as well as knowledge gaps. Findings included beliefs that paediatric patients cannot reliably indicate pain or do not need analgesia. Although the study did posit a correlation between the level of personnel’s qualification and attitudes, the KAP survey method used did not explore the underlying reason for these views (Lourens et al. 2019).

In South Africa, there are no legislated or regulated structured internships or supervised practice programmes to support new graduates during their transition into professional practice. Consequently, emergency care students graduate and are immediately expected to make independent clinical decisions regarding patient care, often with varying levels of early-career supervision.

The absence of mandated and structured transition programmes places the full burden on undergraduate education programmes to prepare graduates for independent, holistic practice. This underscores the need not just for comprehensive and responsible curriculum development but also for effective delivery of curriculum using proven learning and teaching techniques.

Deliberate and scholarly efforts to enhance the undergraduate pain curriculum in South Africa have begun (Matthews 2025). While emergency medical care education in South Africa is regulated by the Health Professions Council of South Africa (HPCSA) through accredited training standards, very limited in-depth qualitative research has examined undergraduate EMS students’ experiences of pain education. Little is known about the self-perceived knowledge and attitudes, as well as self-confidence and readiness, of this population of students.

The aim of this study was to explore the elements influencing senior undergraduate emergency medical care students’ self-reported attitudes, perceived readiness to practise and confidence in managing paediatric pain in South Africa and while identifying self-reported barriers and enablers shaping their approach to paediatric pain management.

## Research methods and design

### Research setting

The study was conducted at a higher education institution in South Africa offering an undergraduate Emergency Medical Care (EMC) programme. Final-year students who were within 2 months of graduation were recruited.

A qualitative descriptive and exploratory design was used to generate a rich, straightforward account of participants’ perceptions and experiences of paediatric pain management. Qualitative description offers a pragmatic and minimally interpretive approach, making it well suited to exploratory research aimed at producing clear, practice-relevant findings (Hall & Liebenberg 2024). This design supports the generation of contextually grounded insights that may inform future research and policy development (Doyle et al. 2020).

Data were analysed through inductive reflexive thematic analysis (Braun & Clarke 2006), which enabled the active generation of themes grounded in the data.

### Population and sample

Purposive sampling was used to select ten students in the final year of study of a four-year Bachelor’s in Emergency Medical Care (BEMC) who were approximately two months from graduation at the time of interview. Final-year BEMC undergraduate students were eligible for inclusion. These students had completed a dedicated paediatric clinical rotation, including exposure to paediatric emergency and intensive care settings, enabling informed reflection on paediatric pain management. Their position at exit level allowed for exploration of attitudes shaped by undergraduate education and clinical experience.

In qualitative descriptive research, sample size is guided by the depth and relevance of data rather than numerical representation. As observed by Sandelowski (2010), the value of this approach lies in generating rich, contextually grounded insights rather than large samples. The selected sample was therefore considered sufficient to address the research question within a rigorous qualitative framework. Data saturation guided the final sample size. Saturation was achieved prior to exhausting the eligible population, with no new codes or themes identified and increasing repetition in the data (Rahimi & Khatooni 2024). Consistent with reflexive thematic analysis, emphasis was placed on depth and richness of data rather than a fixed endpoint (Braun & Clarke 2021, 2022). Saturation was reached iteratively as themes were developed and refined until sufficiently detailed and coherent.

The semi-structured interview guide (see Online Appendix 1) was developed by the researchers, based on the aim and research question. The questions were contextually appropriate to the prehospital field and exit-level under-graduate BEMC students. As the authors used interviews for data collection, all data relating to the study concepts were self-reported. The authors did not objectively measure attitudes, behaviours or practices. Interviews were audio recorded and transcribed. Recruitment was conducted face-to-face. Initial contact with participants was made through a class announcement by the class lecturer and a researcher presentation outlining the study purpose, participation requirements and potential risks and benefits. Information and consent packs were distributed, and interested individuals volunteered their contact details directly to the researcher. The interview guide is contained in the supplementary material (see Online Appendix 1).

### Data collection

The principal researcher conducted semi-structured interviews with senior undergraduate students at a South African university. Participation was voluntary, and interviews were conducted in English either face-to-face or online according to participant preference. Face-to-face interviews took place in a private room on campus, while online interviews were conducted via Microsoft Teams. Interviews lasted approximately 30 min – 45 min and were guided by a semi-structured interview schedule. All interviews were audio-recorded with participant consent and supplemented by field notes. The interviewer maintained an informal and relaxed approach to encourage open discussion.

### Data analysis

Reflexive thematic analysis (RTA) (Braun & Clarke 2006) was employed to identify patterns and generate themes from participants’ narratives, capturing their perceptions and interpretations. An inductive approach with open coding was applied, beginning with descriptive codes that were progressively grouped into categories to allow for the generation of themes. The mind-mapping software FreeMind (2014) was used to construct and visualise a thematic map, supporting the iterative comparison and refinement of codes throughout the analysis process.

The principal researcher conducted the initial coding and theme development, engaging in an iterative and reflective process to identify patterns of meaning across the dataset. To enhance the credibility of the analysis, two additional reviewers independently examined the data and generated codes. Collaborative discussions were subsequently held to explore interpretations and refine the coding framework. Consensus was reached through these discussions, ensuring that the themes were coherent, meaningful and grounded in the data while maintaining the reflexive and interpretative nature of the analysis.

### Trustworthiness

Trustworthiness was guided by the criteria of credibility, dependability, transferability and confirmability as described by Lincoln and Guba (1985). Credibility was enhanced through purposive sampling of participants with relevant experience, use of open-ended interview questions and prolonged engagement with the data through face-to-face interviews, verbatim transcription and inductive thematic analysis. Dependability was supported by a clearly defined research design and systematic data collection and analysis procedures, with oversight provided through proposal review and supervisory engagement throughout analysis. Transferability was addressed by providing detailed descriptions of the study context, methods and participant characteristics, allowing readers to assess applicability to similar populations. Confirmability was ensured through reflexivity, transparency and maintenance of an audit trail, supporting the grounding of findings in the data rather than researcher assumptions.

Member checking was not carried out as it was not deemed congruent with the methodology used, as reflexive thematic analysis positions meaning as interpretive rather than fixed. Consistent with this approach, credibility was supported through reflexivity and a transparent audit trail rather than participant validation.

Suzan-Lynn Smit, female, is an operational emergency care practitioner in the public sector with interests in pain management and prehospital system development with a master’s degree in emergency medical care. This professional background not only informed the study design and facilitated rapport with participants but also necessitated ongoing reflexivity to minimise assumptions related to paediatric pain management practices. No prior supervisory or assessment relationship existed between the researcher and participants. Reflexive awareness was maintained throughout data collection and analysis through memo writing and continuous engagement with the data to support credible and transparent interpretation.

Ryan Matthews is an emergency care practitioner, teacher and academic with a PhD degree in emergency medicine. He has research interests in curriculum development and pain management. Dirk Bester holds a PhD degree in biomedical sciences and is an academic in the field of biomedical sciences with transdisciplinary research interests.

### Ethical considerations

Ethical clearance to conduct this study was obtained from the Health and Wellness Science Research Ethics Committee of the Cape Peninsula University of Technology on 17 October 2022. The ethical clearance number is CPUT/HWS-REC 2022/H21. Participants provided informed consent after a face-to-face explanation of the study’s purpose, potential risks and mitigation strategies. Hard copies of the consent forms were included in the information packets given to the sample population at the initial contact. Electronic study data, including audio recordings, transcripts and coded data, were stored on a password-protected hard drive. The paper-based consent forms were stored in a locked cabinet, with the key only available to the primary researcher. All electronic and paper-based material will be stored for 5 years, after which electronic data will be deleted and paper-based data shredded.

Participation was voluntary, with the option to withdraw at any time without consequence. Identified risks included concerns about negative repercussions from candid feedback, as well as potential emotional distress when discussing patient care experiences. Mitigation measures included ensuring strict anonymity through data de-identification, assigning participant codes, limiting access to data and providing access to counselling services if needed.

## Results

This study was conducted among exit-level emergency medical care students at a South African higher education institution to explore attitudes towards paediatric pain management. Semi-structured interviews were conducted with ten final-year students within two months of graduation. Interviews were audio-recorded, transcribed verbatim and analysed inductively using reflexive thematic analysis. The findings present insights into participants’ beliefs and perceptions, as well as self-reported barriers, enablers, confidence and readiness to manage paediatric pain. Collectively, these findings provide an understanding of preparedness for paediatric pain management and highlight areas for potential improvement in undergraduate education, continuing professional development and prehospital clinical practice.

The authors generated the following themes from the data:

Table 1 depicts the themes and categories generated during data analysis.

**TABLE 1:** Themes and categories generated during data analysis.

Themes	Categories
1. Attitudinal dissonance – Positive beliefs versus scepticism towards paediatric pain expression	1.1. Theoretical understanding of the importance of pain management 1.2. The expression of pain by paediatrics 1.3. Patient assessment 1.4. Own emotions hinder decision making/ thought process
2. Fear and uncertainty – Perceived educational gaps shaped attitudes and perceived readiness	2.1. Perceived confidence and readiness 2.2. Personal attitudes 2.3. Teaching and learning 2.4. Barriers to paediatric pain management 2.5. Enablers to paediatric pain management

### Theme 1: Attitudinal dissonance – Positive beliefs versus scepticism towards paediatric pain expression

A strong theoretical commitment to paediatric pain management was unanimously articulated, framing it as ethically imperative and clinically significant because of its potential long-term physiological and psychological consequences. Strong and emphatic words such as ‘important’ (Participant 4), ‘ethical’ (Participant 7) and ‘detrimental effects’ (Participant 1) were used to describe paediatric pain management. Participants emphasised the importance of early and effective analgesia, noting that patient comfort facilitates accurate assessment and stabilises vital signs. Many acknowledged the prevalence of the insufficient management of prehospital paediatric pain and expressed a personal resolve to avoid contributing to this persistent problem.

This sentiment did not necessarily translate into participants self-reported clinical practices during workplace placements.

Paediatric pain cases were described as emotionally charged and significantly more stressful than adult cases.

Despite having emphatically positive theoretical beliefs on the importance of managing pain in paediatrics, participants were sceptical of the accuracy of paediatric pain presentations. Terms such as ‘exaggerated’, ‘dramatic’, ‘pretend’ and ‘does not translate’ were frequently used to describe children’s pain behaviours.

The dissonance arose in how participants interpreted the way children expressed their pain and the value they assigned to these expressions. While participants recognised the ethical and clinical rationale for intervention, their descriptions of real-world decision-making revealed inconsistent application of these beliefs. Quotes such as:

‘A child’s dramatic pain expression doesn’t really translate to a pain score to me because I believe children cry quite easily so I will use my personal opinion and distraction methods to try to get a pain score.’ (Participant 4)

demonstrate the divorce between the expressed sentiment regarding importance of pain assessment versus the application thereof. This minimisation of expressive behaviours led many to declare that they relied on personal judgement over standardised pain assessment tools or patient self-expression, creating a significant risk of skewed pain assessment.

Participants highlighted the contrast between adult self-reported pain and paediatric pain assessment requiring behavioural tools and clinical judgement. Limited exposure during real-world practice and teaching simulations left students feeling overstimulated, overwhelmed, scared and intimidated, contributing to a sense of unpreparedness in practice. Participants tended to prioritise technical aspects of care, often as a means of reassurance, with advanced monitoring identified as the primary enabler. Support from colleagues and access to a broader range of analgesics also enhanced confidence, particularly through informal consultation and reduced perceived risk in patient management.

The participants reported emotions such as intense stress, anxiety and apprehension in paediatric pain scenarios, which hindered decision-making. These responses, compounded by fear of causing harm and limited experience, contributed to reduced confidence and difficulty managing patient care effectively.

Expressed frustration with emotive behaviours, perceived as obstacles to biomedical and clinical tasks, further highlighted dissonance between the theory of pain management and praxis. These attitudes and frustrations may converge to undermine effective pain management and contribute to oligoanalgesia.

### Theme 2: Fear and uncertainty – Perceived educational gaps shaped attitudes and perceived readiness

In this theme, the participants mostly described negative perceptions and experiences. This was predominantly in relation to components of teaching and learning: (1) content coverage, (2) allocated time and (3) exposure to relevant clinical scenarios:

‘Although the content taught to us may be sufficient, the time spent on the topic is not enough to make us feel prepared and confident. Especially when compared to adult patients. I don’t think we’re ready when we [*will*] graduate.’ (Participant 7)

The preparation for paediatric pain management provided in the undergraduate programme continuously arose as being insufficient and a perceived barrier. Words such as ‘definitely too little’ (Participant 1), ‘way too short’ (Participant 2) and ‘a straight no’ (Participant 5) were used when asked if the undergraduate programme preparation was sufficient in preparing undergraduates for prehospital paediatric pain management.

Participants reported that all paediatric content was compressed into a short block late in the programme, with far greater emphasis placed on adult patient care throughout all years of study. The limited time and abrupt introduction of paediatric topics required students to layer advanced paediatric skills on an adult-focused foundation, leaving them lacking confidence. While participants acknowledged the overall rigour of their undergraduate programme, in respect of pain management, many could not recall any specific paediatric pain content.

Participants reported feeling uncertain, insecure and overwhelmed when managing paediatric patients in pain during workplace learning. This was largely because of limited exposure and insufficient time spent on paediatric content during education and training. Participants reported a lack of confidence and readiness and felt scared and over-stimulated when faced with paediatric pain management in practice. Words used to describe these interactions included ‘I don’t like it, I hate it, I hate it so much’ (Participant 3), ‘More stressed’ (Participant 1), ‘emotionally charged’ (Participant 1) and ‘Less comfortable’ (Participant 7). Communication challenges, such as the limited verbal communication of younger children and age-related linguistic constraints, further compounded these difficulties. This made it hard to de-escalate distressed children and accurately assess pain severity. Emotional and behavioural expressions of pain were described as intimidating and stressful, creating cognitive and affective overload during case management.

Participants experienced multiple difficulties in their provision of sufficient paediatric pain care. These were predominantly clinical task-orientated challenges such as difficult IV access, drug selection and dosage calculations. These were compounded by fear of causing harm or adverse drug reactions. Participants linked these barriers to insufficient time and experience during undergraduate training. Limited workplace learning further exacerbated the problem, although participants emphasised that hands-on exposure and mentorship from senior practitioners were critical for supporting self-confidence and perceptions of competence:

‘My level of readiness is quite low; despite all of my theoretical knowledge I believe only further time and experience will remedy that. Theoretical knowledge can only allow so much confidence and readiness.’ (Participant 10)

When discussing elements that facilitated paediatric pain care, participants again focused on clinical procedures and tasks – such as the use of advanced monitoring devices and the use of non-opioid analgesics, thought to be less risky. Use of advanced monitoring devices and access to a variety of analgesics were perceived to reduce risk and thus provided reassurance. Another identified enabler was professional interaction, ‘practitioners that are willing to pick up the phone’ (Participant 1), offering emotional support and confidence during uncertain situations.

Participants expressed workplace learning as valuable in general but limited with respect to paediatric exposure, as access to paediatric-specific wards only occurred in the fourth year of study. Participants consistently highlighted the positive influence of workplace learning and micro-mentorship from senior practitioners, which provided intellectual stimulation, increased confidence and motivated self-direction.

However, the overall gap in structured paediatric education and the late clinical exposure compromised perceived readiness and self-reported behaviour change.

Participants reported predominantly negative attitudes – rooted in fear and uncertainty – that hindered paediatric pain management. Concerns about incorrect dosing and adverse events, compounded by limited experience and exposure, contributed to hesitancy and reduced confidence in providing safe care.

These two themes illustrate how attitudinal dissonance and fear-driven uncertainty interact with perceived educational gaps to influence confidence, perceived control and decision-making in paediatric pain management.

## Discussion

This research explored the factors influencing senior undergraduate emergency medical care students’ knowledge, attitudes, behaviours, readiness and confidence related to paediatric pain management in the prehospital setting. A central finding was the marked disconnect between students’ theoretical understanding of paediatric pain and their readiness and willingness to manage real-world cases.

Participants consistently described feeling ‘exceptionally low’ in confidence and ‘very uncomfortable’ during paediatric encounters. They cited limited curricular exposure and abrupt, late placement of paediatric content as key contributors. These results mirror findings across health professions, where students report insufficient undergraduate preparation for paediatric pain and analgesia (Dalgarno et al. 2024).

Despite recognising the ethical and clinical importance of early pain management, participants did not appear to translate theory into their reported practice. The limited time allocated to paediatric topics – delivered only late in the programme – and the described dominance of adult-centred teaching meant that students were expected to construct advanced paediatric reasoning on an adult-centric foundation. Descriptions of the way adult content was delivered appeared to match the spiral curriculum model (Merriam & Bierema 2020), which revisits topics iteratively to consolidate complexity. In contrast, paediatric content was delivered suddenly and only once during the programme. Paediatric content was described as lacking scaffolding and contributing to pronounced knowledge gaps and diminished confidence.

Theoretically, participants articulated a theoretical understanding of pain as multidimensional. However, practice-level descriptions indicated a reductionist biomedical interpretation, particularly regarding paediatric pain expression. Discounting children’s emotional and behavioural expressions of pain contradicts contemporary biopsychosocial frameworks (Johnson et al. 2024; Russo 2024). This may also contribute to patterns of delayed care, inaccuracies in assessment and avoidance of analgesia—behaviours consistent with underprepared practitioners navigating complex, high-stake scenarios (Alshehri et al. 2024; Benner 1984).

Participants reported that their limited exposure to paediatric cases during workplace learning compounded these gaps. Although workplace learning and informal micro mentorship from senior practitioners were described as highly valuable, such experiences were limited and inconsistently accessible. The late timing and short duration of clinical placements – combined with minimal structured paediatric exposure – restricted opportunities for integration of theoretical learning, consolidation of clinical reasoning and development of confidence. The authors acknowledge that the participants were still students; however, their proximity to graduation (two months at the time of interview) meant that there was not much time to correct these findings.

The barriers described in our findings – gaps in education, limited exposure and absence of support undermining safe paediatric pain management – have been described elsewhere in prehospital systems (Omotosho et al. 2023; Ozdemir 2019). Descriptions of inadequate undergraduate preparation and potentially inadequate early career supervision and variable organisational support during the transition to independent practice appear systemic in nature (Abdullah et al. 2024; Motsaanaka, Makhene & Ally 2020) and not isolated to our study. This raises concerns for the sufficiency and safety of clinical encounters.

Participants’ attitudes towards paediatric pain played a decisive role in shaping decision making. While participants believed that their theoretical knowledge was adequate, they simultaneously felt unprepared to manage paediatric pain, sufficiently reflecting the well-established relationship between knowledge, attitudes and behavioural intention (Ajzen 1991, 2014). Negative attitudes primarily scepticism towards paediatric pain expression and fears of harming the child combined with limited confidence and perceived lack of control, led to descriptions of hesitation to provide care or even omission of analgesia. Concerns about incorrect drug dosages, side effects and communication challenges further reinforced this reluctance.

The emotional load seemingly carried by participants during paediatric encounters may impede cognitive functioning and contribute to the barriers they described. This pattern aligns with the Dreyfus Model of Skill Acquisition’s Advanced Beginner or Competent stages, where performance is rule based, context recognition is limited, and intuition is underdeveloped (Benner 1984; Dreyfus & Dreyfus 1980). Such performance characteristics are consistent with learners who have limited clinical exposure. Paediatric pain management requires nuanced risk–benefit appraisal, empathic communication and situational awareness—capabilities that depend heavily on experience and cannot be fully developed in the absence of adequate exposure and structured mentorship. In the stated absence of internships, these findings highlight the substantial responsibility placed on undergraduate curricula to prepare graduates for paediatric pain management. Critical observers, however, may ask if this should be the case?

Participants’ descriptions were consistent with international evidence documenting erroneous beliefs among undergraduate learners, such as reliance on vital signs as pain proxies or assumptions that children tolerate pain better than adults (Milani et al. 2025). In this study, such beliefs interacted with low confidence and limited experience, compounding the potential for undertreatment.

Overall, the findings indicate a possible mismatch between curricular intent, teaching delivery and clinical practice needs. Limited paediatric pain content, its late delivery, insufficient experiential learning and inadequate support structures collectively hinder the consolidation of knowledge, the shaping of positive attitudes and the development of behavioural intention conducive to safe, effective analgesia. While technical knowledge provides a foundational base, it must be paired with experience, nontechnical skills and supportive learning environments to foster readiness and confidence.

### Limitations and recommendations

While this research provides valuable insight into the topic of paediatric pain management, there are limitations present. The research did not investigate and explore the curriculum, nor collect data on practice.

These attitudes and perceptions of the participants could thus not be triangulated with objective measures of curriculum and teaching or practice. Participants were senior undergraduate students who, in theory, still had opportunities to further develop their knowledge, skills and attitudes before graduation. Therefore, the findings may not fully represent attitudes at the point of graduation. However, participants were within two months of completing their programme and still needed to begin final assessments. While the interviews were limited to paediatric pain management, the remaining curriculum and examinations covered a broader range of topics beyond paediatrics and pain management. Participants would have thus been dedicating time to these broader topics as well. Considering this context, the authors are confident that the results would not differ substantially if the study had been conducted immediately after graduation.

## Conclusion

This study highlights critical gaps in paediatric pain management readiness among senior EMS students, rooted in undergraduate education and compounded by the absence of structured transition to practice mechanisms. Consistent with positioning at the Advanced Beginner and/or Competent stages of the Dreyfus model, participants expressed a reliance on protocols, limited clinical intuition and difficulty navigating complex paediatric scenarios – capabilities fundamental to safe and ethical care. The persistent theory–practice misalignment, coupled with late paediatric content delivery and insufficient exposure, appears to constrain the internalisation of pain management practice and dampen confidence and attitudes towards its use. To address these deficits, the authors recommend curriculum reform that embraces a design reflective of a spiral curriculum model.

This curriculum might embed simulation and high-fidelity paediatric scenarios to prepare students for the workplace. Targeted workplace placements early in the programme, and formal mentorship to scaffold the transition from classroom knowledge to autonomous professional practice are also recommended. Parallel emphasis on non-technical competencies (communication, situational awareness and cognitive flexibility), reflective practice and decision aids for analgesic selection and dosing under stress is warranted, given evidence that targeted education influences attitudes, intentions and behaviours in clinical contexts (Ajzen 1991; Tran, Nguyen & Kervyn 2018). Taken together, strengthening paediatric pain curricula, instituting structured work-based learning and deliberately cultivating reflective and intuitive capacities are essential steps to reduce oligoanalgesia, enhance clinical reasoning and improve knowledge, confidence and readiness.
